# Vaccination coverage of children aged 12-23 months in Gaziantep, Turkey: comparative results of two studies carried out by lot quality technique: what changed after family medicine?

**DOI:** 10.1186/1471-2458-14-217

**Published:** 2014-03-03

**Authors:** Birgul Ozcirpici, Neriman Aydin, Ferhat Coskun, Hakan Tuzun, Servet Ozgur

**Affiliations:** 1 Department of Public Health, Gaziantep University, Gaziantep, Turkey; 2 Tarsus Community Health Center, Tarsus, Turkey; 3 Ministry of Health, Department of Health Promotion, Ankara, Turkey; 4 The Department of Public Health, Faculty of Medicine, Gaziantep University, 27310 Gaziantep, Turkey

**Keywords:** Family medicine, Fully vaccinated, Gaziantep, Turkey, Immunization, Lot quality technique

## Abstract

**Background:**

Health care systems in many countries are changing for a variety of reasons. Monitoring of community-based services, especially vaccination coverage, is important during transition periods to ensure program effectiveness. In 2005, Turkey began a transformation from a “socialization of health services” system to a “family medicine” system. The family medicine system was implemented in the city of Gaziantep, in December, 2010.

**Methods:**

Two descriptive, cross-sectional studies were conducted in Gaziantep city center; the first study was before the transition to the family medicine system and the second study was one year after the transition. The Lot Quality Technique methodology was used to determine the quality of vaccination services. The population studied was children aged 12–23 months. Data from the two studies were compared in terms of vaccination coverage and lot service quality to determine whether there were any changes in these parameters after the transition to a family service system.

**Results:**

A total of 93.7% of children in Gaziantep were fully vaccinated before the transition. Vaccination rates decreased significantly to 84.0% (p <0.005) after the family medicine system was implemented. The number of unacceptable vaccine lots increased from 5 lots before the transition to 21 lots after the establishment of the family medicine system.

**Conclusions:**

The number of first doses of vaccine given was higher after family medicine was implemented; however, the numbers of second, third, and booster doses, and the number of children fully vaccinated were lower than before transition. Acceptable and unacceptable lots were not the same before and after the transition. Different health care personnel were employed at the lots after family medicine was implemented. This result suggests that individual characteristics of the health care personnel working in a geographic area are as important as the socioeconomic and cultural characteristics of the community.

## Background

Health care systems in many countries are changing, for a variety of reasons. This brings both opportunities and threats for public health professionals. Change offers the possibility to challenge existing arrangements and maximize the contribution of health services to population health. On the other hand, it brings threats as those responsible for health policy seek other objectives, such as the narrow pursuit of profit. Public health professionals, with their emphasis on improving population health, have a legitimate role in ensuring that the pursuit of health gain becomes a central objective of health care systems, whatever other objectives may be being pursued by others. To do so, they must promote the equitable use of interventions that are effective and appropriate for the population in question, reduce interventions that are ineffective or harmful, and thus maximize the health gains obtained with the available funding [1].

A health system described by World Health Organization (WHO) is the sum total of all the organizations, institutions, and resources whose primary purpose is to improve health [2]. In 2005, Turkey began a transformation from a “socialization of health services” system to a “family medicine” system. The pilot system was implemented in Düzce, Turkey, and was introduced throughout the country at the end of 2010. The family medicine system was implemented in the city of Gaziantep, in December, 2010.

The most important main act on health care, the Socialization of Health Services, was adopted in 1961 and its implementation started in 1963 in Muş city. By 1981, 41 cities had socialized health services, and it was declared that the health services were socialized in all cities in Turkey in 1983. The medical services were listed in an hierarchical order from the lowest to the highest as follows: health post, health center, secondary level hospital and tertiary level hospital. The main principle of the socialization of health services was to provide free, equal and permanent health services for all in health care centers where an extensive team of health care professionals worked full-time [3].

In the health transformation programme of Turkey (from a “socialization of health services” system to a “family medicine” system); family physicians are primarily responsible for individuals registered with him/her. Among the duties of the family physician are recording the health records of the persons registered, assuming primary diagnostic and therapeutic services together with immunization and other preventive health care services, and coordinating their secondary and tertiary care. Each family physician will work with a family health staff in the Family Health Center (this staff may be a midwife, a nurse, a health technician, an emergency medicine, technician, a medical secretary or a laboratory technician). Community health centers are located in each subprovince, minimum one in number. They perform public health and administrative services, together with training and supervision activities. By those centers the following tasks are performed in coordination with family physicians: health education of the public, struggle with infectious diseases, preventive environmental health care services, school health services, delivery of equipments for vaccination and family planning and extensive immunization programs, laboratorial, radiological and other diagnostic services, inservice training of the health care staff, forensic medicine, public screenings and collecting medical statistics [4,5].

Vaccine-preventable diseases cause significant morbidity and mortality worldwide and in developing countries in particular. Immunization is an important primary health care in order to prevent infants, children and adults from infectious diseases by injecting before the period having higher risk [6]. WHO indicates that vaccinating children under one year of age against the six vaccine-preventable diseases is one of the most cost-effective programs that protects against illness and death [7]. Turkey started Expanded Programme on Immunization (EPI) in 1981 with 5 vaccines. Today this programme continues with 11 vaccines. According to EPI; “A fully vaccinated child” takes one dose of BCG, three doses of Hepatitis B, 3 doses of mixed vaccine for Diphtheria, Pertussis, Tetanus, inactive Polio, and Heamophilus Influenza type B (Pentac Hib), 3 doses of Pneumococcal Conjugated Vaccine (13v PCV), one dose of Oral Polio Vaccine, 1 dose of Measles, Mumps, Rubella of (MMR) [6]. Hepatitis A and Varicella vaccines are going to be added to schedule in 2013. All routine EPI vaccines are financed by government.

During the transition periods, rapid changes in service areas, and increased staff mobility may disrupt routine services. Therefore, monitoring vaccination coverage becomes more important during these transition periods. The study subjects were children aged 12–23 months that lived in the “lots”, i.e., the provincial districts that were the centers of health care before the system changed to family medicine. The main objective of this study was to establish how routine services are affected by changes in the health care system. The other objectives were to determine vaccination coverage, to determine which lots have a service problem, to compare the results with a study conducted before the transition occurred, and to determine whether conditions have improved. The research results will be shared with administrative authorities to aid in health service planning.

## Methods

This is a descriptive, cross-sectional study conducted in Gaziantep city center by the Lot Quality (LQ) technique. Data were obtained via two studies applied at the same lots. The first study was applied just before transition and the second was applied one year after transition to family medicine.

A representative sample was selected by the LQ technique to decide whether one or more health service units is meeting the standard of performance and to measure vaccination coverage. The population of Gaziantep was 1.244.000. The total number of children aged 12-23 months was (target population) 31.892. The level of accuracy was tested as ±3%, level of confidence as 95% and the total sample size was estimated as 1066. The total number of lots in Gaziantep city center to be studied was 50, so the minimum lot sample size was estimated as 20. The addresses of children aged 12-23 months living in each lot (health center district) were accessed from Turkish Statistics Institute. Twenty original and 5 alternate child was chosen by simple random sampling technique for each lot.

A *threshold* is a percentage used to assess the performance of a lot. It is a level of performance used to judge whether a lot is acceptable or not acceptable [8]. Low threshold level was set as 85% for fully vaccination coverage. A *decision value* is the highest number of individuals in a lot that you can find to be *not* receiving a service and yet still classify the lot as acceptable [8]. So decision value was selected as 3 children (who were not fully vaccinated) in 20 children (%15) for each lot. If we found one more individual than the decision value, we judged the lot “unacceptable”. In this study, state of being fully vaccinated was evaluated as mentioned on EPI [6].

WHO’s book for LQ technique was used as guideline for preparing the questionnaire [8]. This questionnaire was applied to mothers by intern doctors and researchers between December 1-31 2010 in the first study and between December 1-31 2011 in the second by face to face technique. Informed consent for participation in the study was obtained from mothers. We asked whether the child had vaccination card, whether the child was vaccinated, if yes, vaccination dates (if the child is vaccinated at the appropriate age or not), subsequent doses are given after an appropriate interval, where the child was vaccinated, and if not, we asked why they did not. For Bacille Calmette Guerin (BCG), we examined if he or she had a BCG scar (Additional file 1).

Lot quality assurance sampling, otherwise known as the LQ technique, is a quality control tool adopted from industry and is based on collecting important management information by using small-size samples randomly taken from specific service and settlement units. LQ technique is a quick and easy method that can be used by healthcare managers at all levels to monitor and evaluate health programs. In healthcare, the sample is usually taken from a group of people that constitute the ‘lot’ [9].

It is designed to identify health centres or other health service units that are not meeting coverage targets or other standards, so that attention can be directed to the units most in need. Advantages of the LQ technique are; to make judgements about individual health service units (i.e., lots), allowing managers to direct supervision and other resources to the units that need it most, to interpret data as soon as they are collected from a health service unit. In addition to its capabilities as an assessment and supervision tool, the LQ technique can be used to survey coverage. The LQ technique has two important limitations: coverage in individual health service units can be judged in a general sense as acceptable or not acceptable, but specific levels of coverage can be calculated only for all of the units in the study. Selecting a lot sample size and a decision value involves the assessment of risks. The risk to the service provider is that resources will be spent on relatively good health service units because they have been wrongly identified as unacceptable. The consumer or client risk is that real health service problems will be wrongly identified as acceptable and nothing will be done to improve them [8].

This technique was used in a good many studies and was proved useful in identifying small health areas with lower vaccination coverage, easy for staff to use, feasible for routine monitoring of vaccination coverage [10-12]. Training of local health personnel on use of the LQ technique could expedite response to local health problems and could even motivate them in conducting their own surveys tailored to their professional interests [13].

The data were analyzed in SPSS and one sample test for comparing proportions was used.

### Ethics statement

Gaziantep University ethics committee has confirmed that this study would not have required ethics approval.

## Results

Results for vaccine coverage for the first and second study are presented in Table 1. In the text, the results of the first study were given bold for comparison.

**Table 1:** Comparative results of the two studies

	**The first study (n = 1000)**		**The second study (n = 1000)**	
	**Just before transition to family medicine**		**One year after transition to family medicine**	
	**n**	**Coverage (%)**	**n**	**Coverage (%)**
**BCG**	989	98.9	985	98.5
**BCG Scatris +**	982	98.2	972	97.2
**Pentac-Hib 1**	989	98.9	991	99.1
**Pentac-Hib 2**	983	98.3	975	97.5
**Pentac-Hib 3**	976	97.6	956	95.6
**Pentac-hib booster**	599	59.9	652	65.2
**Oral polio 1**	978	97.8	938	93.8
**Oral polio 2 booster**	597	59.7	630	63.0
**Hepatititis B 1**	990	99.0	998	99.8
**Hepatititis B 2**	987	98.7	986	98.6
**Hepatititis B 3**	977	97.7	954	95.4
**MMR**	974	97.4	920	92.0
**13v PCV 1**	983	98.3	986	98.6
**13v PCV 2**	977	97.7	970	97.0
**13v PCV 3**	970	97.0	952	95.2
**13v PCV booster**	926	92.6	883	88.3
**Fully vaccinated**	937	93.7	840	84.0

Of the children that participated in each study, 83.2% (results for 2nd, post-transition, study) vs. **56.0%** (results for the 1st, pre-transition, study) had a vaccination card. Data on vaccination was obtained via the vaccination card for 49.0% vs. **36.5%,** via anamnesis for 17.0% vs. **44%,** and by both vaccination card and anamnesis for 34.0% vs. **19.5%** of the study subjects.

Coverage for the BCG vaccine was 98.5% vs. **98.9%,** and the subjects were most often vaccinated at family health centers (or, “health centers”; 99.7% vs. **98.2%)**.

The first dose of the Pentact-HIB vaccine was given to 99.1% vs. **98.9%** of children and they received the vaccine at family health centers 99.9% vs. **97.8%** of the time. Coverages for the second, third, and booster doses of the Pentac-Hib vaccine were 97.5% vs. **98.3%,** 95.6% vs. **97.6%**, and 65.2% vs. **59.9%,** respectively.

The first dose of oral polio vaccine was given to 93.8% **(97.8%)** of children. The coverage was 63.0% **(59.7%)** for the booster dose of oral polio vaccine.

Vaccine coverage for the first, second, and third doses of Hepatitis B vaccine were 99.8% **(99.0%)**, 98.6% **(98.7%)**, and 95.4% **(97.7%)** respectively. The first dose was usually given at hospitals (76.0%; **28.6%)**. The second and third doses were most often given at family health centers (98.9% **(99.6%)** for the second dose; 97.0% **(97.6)** for third the dose.

Coverage for the MMR vaccine was 92.0% **(97.4%)**, and this vaccine was given at family health centers 99.9% **(98.3%)** of the time.

The coverage for the first dose of PCV was 98.6% **(98.3%)**. The coverage for the second, third, and booster doses were 97.0% **(97.7%)**, 95.2% **(97.0%)**, and 88.3% **(92.6%)**, respectively; this vaccine was usually given at family health centers.

Full vaccination was achieved for 84.0% **(93.7%)** of children. The difference between the percentage that were fully vaccinated before the transition compared with after the transition is statistically significantly different (p < 0.005); i.e., the percentage of children fully vaccinated after the transition was much lower than before the transition. The coverage for full vaccination increased to 85.64% **(93.62%)** when weighted according to population size. However, this value was also significantly lower in the second study compared with the first study (p < 0.005).

Of the children that were not fully vaccinated, 15.4% **(5.4%)** received some but not all of the vaccines and 0.6% **(0.9%)** of them were never vaccinated.

Twenty-one of the 50 lots were judged as unacceptable in terms of achievement of vaccination goals in the second study. The number of unacceptable lots was **5** in the first study.

The mothers of children who were incompletely vaccinated or were never vaccinated gave the following reasons for non-vaccination of their children: “I don‘t know when my child will be vaccinated” (11.8%); “family problems” (8.7%); “child was ill” (8.7%); and “rumors on vaccines” ( some people said bad things about vaccines) (7.9%).

## Discussion

Achievement and maintenance of high vaccination coverage for vaccine-preventable diseases is an important part of programs to achieve control, elimination, or eradication of these diseases. Vaccination cards are considered a quality measure in vaccination services and are extremely important to obtain information about vaccination history. Of the children that participated in this study, 83.2% **(56.0%)** had a vaccination card. Registration increased after the family medicine system was implemented. Because family physicians are being audited (investigated, checked out) via computer and because they take performance fees for their services, records might have become better. However, registration is still a problem in Gaziantep although it is an urban region. It is possible that mothers lost their child’s vaccination cards. According to the countrywide Turkish Demographic and Health Survey, 2008 (TDHS 2008), 75.8% of 12–23 month old children had a vaccination card [14]. In a study performed by LQ technique in Ankara (the capital of Turkey), only 57.5% of parents/caregivers could present the card when asked in the interviews [13]. In another study from rural Kenya, vaccination cards were available for 86% of children [15]. Health workers’ attention about records, and familial concern about vaccination and keeping vaccination cards should be increased by health education.

Tuberculosis is a re-emerging problem around the world. Neonatal BCG vaccine is safe and effective, with an overall protective value of 75% [16]. Worldwide, different coverage rates have been obtained for this vaccine. BCG vaccine coverage was 98.5% **(98.9%)** in our study. In a study we conducted in the South-East Anatolia region (SEAP) of Turkey ten years ago (it was carried out between 2001 and 2002 and Gaziantep is a city in SEAP), BCG vaccine coverage was 76.7% in children aged 12–23 months [17]. In the last ten years, good progress has been achieved for BCG vaccine coverage. The small decrease revealed by the second study may be temporary, and due to the transition period. In the TDH 2008 survey, BCG coverage was 95.9% for the entire country [14]. In a study performed by LQ technique in Ankara in 2006, coverage for BCG vaccine was 99.4% [13]. In Nepal, BCG coverage was 96.7% [18]. In Dinghai, China, the timely BCG coverage rate was 22.26% [19]. In the UK, of 5308 infants born in 2003, 514 (9.6%) were at risk for TB; 423 (82.2%) of these infants were referred postnatal for BCG vaccination, and 391 received it [16]. Coverage of BCG was 89.3% in a rural South African population [20].

Of the children in this study, 99.1% **(98.9%)** received their first dose of Pentac-Hib vaccine. The coverage was 95.6% **(97.6%)** for the third vaccine dose. In the SEAP, coverage was 62.0% for the third dose of the Diphtheria, Tetanus, Pertussis (DPT) vaccine [17]. Coverage results from our two studies indicate that good progress for coverage of the third dose has been achieved in the past ten years. In a study performed by LQ technique in Ankara coverage for three doses of DPT was 98.5% in 2006 [13]. The TDH 2008 survey indicated that, in children 12–23 months of age, coverage for DPT 1 was 96.6%; for DPT 3 coverage was 85.9% for the entire country [14]. In Dinghai, the timely coverage rate of DPT was 91.40% [19]. In Kenya, 95% of children received three doses of the DTP-HepB-Hib vaccine [15]. In Nepal, 90.0% coverage was achieved for DPTHb-3 [18].

In this study, 93.8% **(97.8%)** of children received the first dose of oral polio vaccine. The coverage was 63.0% **(59.7%)** for the second dose. In the SEAP ten years ago, 62.0% received the third dose of oral polio vaccine [17]. In the TDH 2008 survey, Polio1 coverage was 95.8%, Polio2 coverage was 92.3%, and Polio3 coverage was 86.3% in children 12–35 months of age [14]. Our data indicate that, compared with the TDH 2008 survey, coverage for Polio2 was lower in Gaziantep, before and after the transformation. Because children receive inactive Polio vaccine in Pentac-Hib vaccine before, this may lead to neglect of second oral polio vaccine. In Nepal, the coverage for Polio3 was 97.6% [18]. In Dinghai, the timely coverage rate of OPV was 90.82% [19].

In this study, coverage for the third dose of Hepatitis B vaccine was 95.4% **(97.7%)**. In the SEAP ten years ago the coverage was 44% for the third dose of Hepatitis B vaccine [17]. Good progress has been achieved in ten years. In a study performed by LQ technique in Ankara coverage for three doses of hepatitis B was 97.3% in 2006 [13]. The coverage rate was 84.7% in the TDH 2008 survey for the third dose of Hepatitis B vaccine [14]. In Dinghai, the timely coverage rate of Hepatitis B was 95.02% [19]. A Hepatitis B seroepidemiology study of ten European countries revealed that the seroprevalance of antibodies was lower than the reported in three countries [21]. Higher vaccination coverage should be obtained to achieve targeted antibody seroprevalence levels.

As measles is a highly infectious disease, the UK recommendation is for at least 95% of children to receive a first vaccination with the MMR vaccine before age 2 years and a booster before age 5 years to achieve herd immunity and prevent outbreaks [22]. The coverage of the MMR vaccine was 92.0% **(97.4%)** in our study. In the SEAP ten years ago, coverage for the measles vaccine was 62.7% [17], which indicates that good progress is being made. Prevalence of having at least one dose of vaccination against measles was 93.9% in Ankara [13]. The TDH 2008 survey revealed that coverage was 85.8%, countrywide [14]. In Nepal, the coverage for measles was 78.1% [18]. In Dinghai, the timely coverage rate of the measles vaccine was 95.40% [19]. In a rural South African population, coverage of measles was 77.3% [20]. In a rural area in Kenya, 88% of children received the measles vaccine [15]. An elimination program in the WHO European Region aimed to achieve and maintain a coverage of 95% for two doses of MMR. Despite strong health care systems in European countries, in recent years this region has experienced an outbreak of measles. In fact, one of the largest outbreaks in the world occurred in Europe [23]. Similar outbreaks by an incidence of 1/100.000 (according to WHO criteria, a higher incidence of elimination) was occurred not only in France; but also in other European countries; Bulgaria, Ireland and Switzerland. Germany and Greece were also influenced by the outbreak [24]. In a study in India by LQ technique, coverage of measles vaccine was 97.7% [11]. In this globalized world, it is not enough to reach the coverage goals in one country or area; all countries should achieve them. Imported cases are an important problem that all countries should address.

In this study, the coverage of the first PCV dose was 98.6% **(98.3%).** The second, third, and booster doses were 97.0% **(97.7%)**, 95.2% **(97.0%)**, and 88.3% **(92.6%),** respectively. Because this vaccine was added to the schedule in recent years, there were no studies available for comparison.

The timeliness of children’s vaccination varies widely between and particularly within countries, and published yearly estimates of national coverage do not capture these variations. Delayed vaccination could have important implications for the effect of new and established vaccines on the burden of disease [25]. In our study, 84.0% of children were fully vaccinated after the transition to the family medicine system, which was significantly lower than before the transition **(93.7%)** (p < 0.005). When calculated as weighted to the population, full vaccination coverage increased to 85.64% **(93.62%)**. In the SEAP ten years ago, only 30% of children had received all required vaccines [17], so major progress has been achieved in ten years. In the TDHS 2008 study, coverage was 74.9% for the entire country [14]. In Istanbul, Turkey, the completed vaccination rate was 84.5% [26]. The coverage of fully vaccinated children was 75.1% in Edirne and was 88.9% in Bolu by LQ technique studies performed in the second year of the transition. These cities in western Turkey began the transition to the family medicine system before Gaziantep City [27,28]. In the EPI of Turkey, the main objectives are to achieve 95% coverage for each vaccine in the schedule, to maintain coverage throughout the country, and to achieve full immunization for 90% of children 12–23 months of age [6]. The coverage of fully vaccinated children is under the goal of EPI all in all provinces (Gaziantep, Bolu and Edirne Provinces). In another study by LQ technique in Ankara 91.3% of participants were fully vaccinated in 2006 [13]. In a study in İstanbul by the same method, 75.6% of the children aged 12–23 months were fully vaccinated in 2001 [9]. However, it should be kept in mind that studies had been conducted in different years and different threshold levels were used. In a LQ technique study, full vaccination of children in India was 84.09% [29]. In a study from rural Nigeria, a cluster survey study revealed that vaccination coverage against the seven childhood vaccine preventable diseases was 61.9% [30]. In a study in Nairobi, up-to-date coverage with all vaccinations at 12 months of age was 41.3% [31]. According to the results of 2009 national survey in Haiti, 40.4% of children had received the eight recommended vaccinations [32]. Worldwide, more progress is needed on achievement of full vaccination coverage.

The number of unacceptable lots increased from 5 lots to 21 lots after the transition to family medicine. This result indicates that >15% of children in these lots were not fully vaccinated. If we take the EPI criterion (>90%) as threshold [6], then fewer lots are meeting this goal. The specific lots that were classified as acceptable or unacceptable were different before and after the transition. Individual characteristics of the health care personnel working in an area may be as important for lot quality as the socioeconomic and cultural characteristics of the community. Because different health care personnel were working at the lots after the family medicine system was established. In addition, it is too early to say, type of health care system may affect this.

Information on coverage and reasons for non-vaccination is vital for the improvement of vaccination programs. The mothers mentioned the following reasons for non-vaccination of their children: “I don‘t know when I will take my child to be vaccinated” (11.8%); “family problems” (8.7%); “child was ill” (8.7%); and “rumors on vaccines ” (7.9%). These reasons are similar to the reasons recorded in other studies and seemed to be related with incorrect knowledge and educational status. The reasons reported for non-vaccination in a study in Ankara were “being unaware of a need for vaccination”, “not knowing that a subsequent dose is also needed”, “being away from home/area at the time for vaccination” and/or “familial reasons” [13]. In Nigeria, completeness of vaccination was significantly correlated with a mother’s knowledge about immunization [30]. In Haiti, reasons for under-vaccination included insufficient time to reach the vaccination location (24.8%), having a child who was ill (13.8%), and not knowing when, or forgetting, to have the child vaccinated (12.8%) [32]. One frequently reason found in all studies was “the child was ill”. It is important to educate parents about accurate reasons for non-vaccination. The use of mass media may help in such activities and qualitative researches on reasons for non-vaccination may be helpful to understand the problem in-depth.

More attention should be given to public education if high coverage levels are to be achieved and maintained. Health personnel should focus on vaccination during their health education activities; they should also encourage mothers not to lose vaccination cards. Efforts to improve the immunization program should include training for vaccination staff to encourage initiative, so that missed opportunities will be minimized.

## Conclusions

Vaccination coverage declined after the transition to a family medicine system in Gaziantep, Turkey, and there was an increase in the number of unacceptable lots. These declines may be temporary and due to the transition; however, it is important to continue monitoring to determine whether coverage improves.

In the EPI of Turkey, the main objectives are to achieve 95% vaccination coverage for each vaccine in the schedule and to fully immunize >90% of children 12–23 months of age [6]. In the second study, the target was achieved for the BCG, Hepatitis B, PCV, and Pentac-Hib vaccines, but not for the OPV and MMR vaccines. The goal for full vaccination have not been reached. However, the fully vaccinated coverage was above the target a year before the transformation. Because the health care system cannot ensure that a child will receive the required vaccinations, if he or she is not registered at a family physician’s list. The target population of a lot is likely to be larger than indicated by the public health records. The District Health Office coverage reports were remarkably higher than the coverage of immunizations obtained by this study, which indicated that there were more children in the population that needed vaccination.

The coverage for first doses of vaccine was higher after the transition than before the transition. However, second, third, and booster doses and the number of children that were fully vaccinated were lower after the transition. This difference may be due, first, to family migration (the migration rate is higher in some areas) combined with no change to a new family physician. Second, in the previous system (Socialization of Health Services), a child could be vaccinated as a “guest” at any health center; but “guest” vaccination is more difficult to achieve in the family medicine system. Third, administrative authorities request that family physicians use computerized vaccine barcodes so that there is an online record for each child’s vaccine status. This practice avoids vaccine waste, but may inhibit family physicians from using initiative and vaccinating a child who is not in their records. Finally, it is possible that the rate of booster vaccination is lower because, over time, physicians and families lose interest in maintaining vaccine coverage.

Following studies by LQ technique will be necessary to evaluate whether vaccine coverage increases and evaluate how community-based services are being affected by the family medicine system.

## Abbreviations

WHO: World Health Organization; EPI: Expanded Programme on Immunization; BCG: Bacille Calmette Guerin; Pentac Hib: mixed vaccine for Diphtheria, Pertussis, Tetanus, inactive Polio, and Heamophilus Influenza type B; 13v PCV: Pneumococcal Conjugated Vaccine; MMR: Measles, Mumps, Rubella; TDHS 2008: Turkish Demographic and Health Survey 2008; SEAP: South-East Anatolia Project Region; LQ: Lot Quality technique.

## Competing interests

The authors declare that they have no competing interests.

## Authors’ contributions

BO, NA: have made substantial contributions to conception and design, acquisition of data, analysis and interpretation of data; have been involved in drafting the manuscript and revising it critically for important intellectual content; and have given final approval of the version to be published. FC, HT: have made substantial contributions to conception and design, acquisition of data, and analysis and interpretation of data; have given final approval of the version to be published. SO: have made substantial contributions to conception and design, revised it critically for important intellectual content; have given final approval of the version to be published.

## Pre-publication history

The pre-publication history for this paper can be accessed here:

http://www.biomedcentral.com/1471-2458/14/217/prepub

## Supplementary Material

Additional file 1 Infant Immunization Questionnaire. Click here for file
