# Supplementary material for: Vaccination coverage of children aged 12-23 months in Gaziantep, Turkey: comparative results of two studies carried out by lot quality technique: what changed after family medicine?
Source: BMC Public Health. 2014 Mar 3;14:217. doi: 10.1186/1471-2458-14-217 (PMC4015608; doi:10.1186/1471-2458-14-217)
Supplement: Additional file 1 — Infant Immunization Questionnaire. [file 1471-2458-14-217-S1.doc]

**Infant Immunization Questionnaire**

Lot number:

Date:

Town:

Name of Lot:

Number of Child in lot:

-Name and Surname of Child:

-Birth Date:

-Immunization card ?

1)Yes 2)No

**BCG Vaccine;**

1)Yes 2)No

BCG Scar?

1)Yes 2)No

Vaccination Date:

Source:

( Health Center, Tuberculosiz Struggle Center.,Hospital, Private Doctor,etc.)

**Pentac Hib Vaccine :mixed vaccine for Diphtheria, Pertussis, Tetanus, inactive Polio, and Heamophilus Influenza type B.**

| First dose date: | Second dose date: | Third dose date: | Booster date: |
| --- | --- | --- | --- |
| First dose source: | Second dose source: | Third dose source: | Booster source: |

**OPA (Oral Polio Vaccine)**

| First dose date: | Second dose date: |
| --- | --- |
| First dose source: | Second dose source: |

**Hepatitis B Vaccine**

| First dose date: | Second dose date: | Third dose date: |
| --- | --- | --- |
| First dose source: | Second dose source: | Third dose source: |

**Measles, Mumps, Rubella Vaccine (MMR)**

| First dose date: |
| --- |
| First dose source: |

**Pneumococcal Conjugated Vaccine (13v PCV)**

| First dose date: | Second dose date: | Third dose date: | Booster date: |
| --- | --- | --- | --- |
| First dose source: | Second dose source: | Third dose source: | Booster source: |

-What is the source of information on immunization?

1)Only immunization card

2) Only mother

3) Both mother and immunization card

- Immunization status of child?

1)Never vaccinated

2)Partially vaccinated

3) Fully vaccinated

-Fully immunized before one year of age?

1. Yes (Thanks for your anwers)
2. No

**- Reasons for Immunization Failure**

( If mother mentions more than one reason give number begining from the most important reason).

( ….) Unaware of need for immunization

( ….) Unaware of need to return for next dose

( ….) Place of immunization unknown

( ….) Time of immunizationunknown

( ….) Fear of side effects

( ….) Fear of injections

( ….) Rumors (specify)………………………………………

( ….) Place of immunization too far away

( ….) Time of immunization inconvenient

( ….). Vaccinator absent

( ….) Vaccinator told mother that child was already fully immunized

( ….) Vaccine not available

( ….) Parent too busy

( ….) Family problem, including illness of mother…………………………………

( ….) Child ill - not brought

( ….) Child ill - brought but not given immunization

( ….) Long waiting time

( ….)Other…………………………………………………

Name of intervieweer: Signature:
